# Characterization of Malnutrition in Atrial Functional Mitral Regurgitation

**DOI:** 10.1016/j.cjco.2025.05.007

**Published:** 2025-05-21

**Authors:** Tsukasa Murakami, Nobuyuki Kagiyama, Tomohiro Kaneko, Kazuki Kagami, Masashi Amano, Taiji Okada, Yukio Sato, Yohei Ohno, Kimi Sato, Kojiro Morita, Tomoko Machino-Ohtsuka, Yukio Abe, Hideki Ishii, Masaru Obokata

**Affiliations:** a Department of Cardiovascular Medicine, Gunma University Graduate School of Medicine, Maebashi, Japan; b Department of Cardiology, Japanese Red Cross Ogawa Hospital, Saitama, Japan; c Department of Cardiovascular Biology and Medicine, Juntendo University Graduate School of Medicine, Tokyo, Japan; d Department of Heart Failure and Transplantation, National Cerebral and Cardiovascular Center, Suita, Japan; e Department of Cardiovascular Medicine, Kobe City Medical Center General Hospital, Kobe, Japan; f Department of Cardiology, St Marianna University School of Medicine, Kanagawa, Japan; g Department of Cardiology, Tokai University School of Medicine, Isehara, Japan; h Department of Cardiology, Institute of Medicine, University of Tsukuba, Tsukuba, Japan; i Department of Nursing Administration and Advanced Clinical Nursing, Division of Health Sciences and Nursing, Graduate School of Medicine, The University of Tokyo, Tokyo, Japan; j Department of Cardiology, Osaka City General Hospital, Osaka, Japan

**Keywords:** malnutrition, heart failure, atrial functional mitral regurgitation, Geriatric Nutritional Risk Index

## Abstract

**Background:**

In this study we sought to characterize the prevalence, clinical characteristics, and outcomes of malnutrition in patients with atrial functional mitral regurgitation (AFMR).

**Methods:**

This multicentre, observational study included 802 patients diagnosed with AFMR. The **G**eriatric **N**utritional **R**isk **I**ndex (GNRI) was used as a nutritional risk metric. Patients were divided into 4 groups on the basis of the GNRI: normal (> 98; n = 342), mild nutritional risk (92-98; n = 196), moderate risk (82 to < 92; n = 166), and severe risk (< 82; n = 98). The primary outcome was a composite of heart failure admission and all-cause death.

**Results:**

At least mild nutrition risk (GNRI ≤ 98) was present in 57% of patients with AFMR. Patients with lower GNRI were older, had lower body mass index, hemoglobin levels, and renal function, and had a higher prevalence of New York Heart Association class III or IV, dementia, and impaired activities of daily living. During the median follow-up duration of 978 (interquartile range, 492-1141) days, 254 primary outcomes were observed. Increasing severity of malnutrition risk categories was associated with higher rates of the primary outcome. Multivariable analysis revealed that a continuous metric of GNRI was associated with the primary outcome after adjusting for multiple confounders (adjusted hazard ratio, 0.76 per 1 standard deviation increment; 95% confidence interval, 0.66-0.87; *P* < 0.01). Follow-up GNRI values were available in 234 patients (29.2%). Patients with a decreased GNRI over time had higher rates of the composite outcome than those with preserved GNRI (adjusted hazard ratio, 3.83; 95% confidence interval, 1.97-7.43; *P* < 0.01).

**Conclusions:**

Patients with AFMR and malnutrition represent a vulnerable population with worse clinical outcomes.

## Résumé

**Contexte:**

Cette étude visait à caractériser la prévalence, les caractéristiques cliniques et les conséquences de la malnutrition chez des patients atteints de régurgitation mitrale fonctionnelle auriculaire (RMFA).

**Méthodologie:**

Cette étude multicentrique observationnelle comprenait 802 patients ayant reçu un diagnostic de RMFA. L’indice GNRI (*Geriatric Nutritional Risk Index* – indice évaluant le risque nutritionnel chez les personnes âgées) a été utilisé pour mesurer le risque nutritionnel. Les patients ont été répartis en quatre groupes selon leur score GNRI : état nutritionnel normal (> 98, n = 342), risque nutritionnel léger (92 à 98, n = 196), risque nutritionnel modéré (82 à < 92, n = 166) et risque nutritionnel élevé (< 82, n = 98). Le paramètre d’évaluation principal combinait l’admission à l’hôpital pour insuffisance cardiaque et le décès toutes causes confondues.

**Résultats:**

Un risque nutritionnel à tout le moins léger (GNRI ≤ 98) a été observé chez 57 % des patients atteints de RMFA. Les patients ayant un score GNRI plus bas étaient plus âgés, présentaient un indice de masse corporelle, un taux d’hémoglobine et une fonction rénale plus faibles, et une prévalence plus élevée de l’insuffisance cardiaque des classes III et IV de la NYHA, de la démence et de la détérioration de la capacité à accomplir les activités de la vie quotidienne. Pendant la période de suivi médiane de 978 jours (intervalle interquartile : 492 à 1 141), le paramètre d’évaluation principal a été observé à 254 reprises. Plus le risque de malnutrition augmentait et plus le taux de survenue du paramètre d’évaluation principal était élevé. L’analyse multivariée a montré que la mesure continue du score GNRI était associée au paramètre d’évaluation principal après ajustement pour tenir compte de plusieurs facteurs confusionnels (rapport des risques instantanés [RRI] ajusté de 0,76 par incrément d’un écart-type; intervalle de confiance [IC] à 95 % : 0,66 à 0,87; *p* < 0,01). Les valeurs de suivi du score GNRI étaient connues pour 234 patients (29,2 %). Les patients dont le score GNRI a diminué au fil du temps présentaient des taux plus élevés de survenue du paramètre composite, comparativement à ceux dont le score GNRI s’est maintenu (RRI ajusté : 3,83; IC à 95 % : 1,97 à 7,43; *p* < 0,01).

**Conclusions:**

Les patients atteints de RMFA et de malnutrition constituent une population vulnérable au pronostic clinique moins favorable.

Atrial functional mitral regurgitation (AFMR) is increasingly recognized as a distinct subtype of mitral regurgitation (MR).^1^, ^2^, ^3^, ^4^ Unlike ventricular functional MR,^5^ AFMR is caused by mitral valve (MV) leaflet malcoaptation from the stretch of the mitral annular and left atrium.^6^ Despite advances in the understanding about its mechanisms,^7^ clinical characteristics in patients with AFMR have been largely unknown. Malnutrition is common in patients with various cardiac diseases, such as heart failure (HF),^8^, ^9^, ^10^ atrial fibrillation (AF),^11^ coronary artery disease,^12^ and valvular heart diseases,^13^, ^14^, ^15^ and is associated with worse clinical outcomes.^8^, ^9^, ^10^, ^11^, ^12^, ^13^, ^14^ Because of the high prevalence of AF and HF with preserved ejection fraction (HFpEF),^6^^,^^16^ malnutrition would be highly prevalent in patients with AFMR and would be associated with poor outcomes. Accordingly, in this this study we sought to characterize the prevalence, clinical characteristics, and clinical outcomes of malnutrition in patients with AFMR.

## Methods

This study was a retrospective analysis of the **RE**al-world obser**V**ational study for inv**E**stig**A**ting the preva**L**enceand therapeutic options for **A**trial **F**unctional **M**itral **R**egurgitation (REVEAL-AFMR).^4^ The REVEAL-AFMR was a retrospective observational study involving patients with ≥ moderate AFMR in Japan. The design and primary findings have been published elsewhere.^4^ In brief, a total of 177,235 patients who underwent transthoracic echocardiography (TTE) between January 2019 and December 2019 at 26 participating centres in Japan were retrospectively screened to identify patients with age 20 years and older and moderate or greater MR. AFMR was defined as MR without degenerative changes in the MV, with a preserved ejection fraction of ≥ 50% and a dilated left atrium (left atrium volume index ≥ 38 mL/m^2^ for men and ≥ 41 mL/m^2^ for women; if the left atrial volume index was not available, left atrial diameter ≥ 40 mm for men and ≥ 37 mm for women was used). Patients with MR due to systolic anterior motion of the MV, MR subsequent to MV surgery, and acute decompensated HF were excluded. A total of 50 randomly selected echocardiographic images were checked in the imaging core laboratory to confirm the reliability of diagnosis, severity of regurgitation, and values of the measurements.

From the cohort of the REVEAL-AFMR, patients with serum albumin levels measured within 90 days of the baseline date were identified. The REVEAL-AFMR was approved by the IRB of the Juntendo University, Japan, with each participating centre approving the execution of the study (UMIN000046146), and was performed in accordance with the Declaration of Helsinki.

### Assessment of malnutritional risk

The **G**eriatric **N**utritional **R**isk **I**ndex (GNRI) is an objective tool to estimate the risk of malnutrition-related complications in older adults.^17^ The GNRI was calculated as follows: 14.89 × serum albumin levels in g/dL + 41.7 × (actual body weight in kg/ideal body weight in kg). If the ratio of actual body weight to ideal body weight was greater than 1.0, the value was set to 1.0. The ideal body weight was calculated by using the Lorentz formula as follows: height in cm - 100 - (height in cm - 150)/4 for men and height in cm - 100 - (height in cm - 150)/2.5 for women. Patients were then classified on the basis of the GNRI value: normal, > 98; mild risk, 92-98; moderate risk, 82 to < 92; and severe malnutrition risk, < 82. Follow-up GNRI was also calculated if serum albumin levels were measured within 90 days of follow-up assessment. Because of missing height data at follow-up, calculations were made assuming the same height as at baseline. **Co**ntrolling **Nut**ritional Status (CONUT) scores and **P**rognostic **N**utritional **I**ndex (PNI) were also calculated.^18^^,^^19^ The CONUT score was calculated by summing designated points of 3 laboratory markers (serum albumin, total lymphocyte count, and total cholesterol).^18^ CONUT score ranges from 0 to 12, and scores of 0-1 were classified as normal, 2-4 as mild risk, 5-8 as moderate risk, and 9-12 as severe risk. The PNI was calculated as follows: 10 × serum albumin in g/dL + 0.005 × total lymphocyte count per μL, and a PNI > 38 was classified as normal, 35-38 as moderate risk, and < 35 as severe risk.^19^ PNI does not have a risk category of “mild risk.”

### Echocardiography

Echocardiography was performed in all patients and interpreted according to the current guidelines.^20^ Concentric hypertrophy was defined as relative wall thickness (RWT) > 0.42 and left ventricular mass index > 115 g/m^2^ for men and > 95 g/m^2^ for women.^21^ The details of the calculation of RWT and left ventricular mass index were previously described.^21^ MR severity was evaluated using the guideline-recommended comprehensive approach.^22^

### Outcome data

Participants were followed up, and data on a composite outcome of HF admission and all-cause death were collected. Telephone surveys were conducted with patients or relatives to confirm end points if more than 1 year had elapsed since the patient’s last follow-up visit.

### Statistical analysis

Data are presented as percentages for categorical variables, mean ± standard deviation for normally distributed continuous variables, or median and interquartile range (IQR) for non-normally distributed continuous variables. Categorical variables were compared using Fischer exact test. Continuous variables were compared using 1-way analysis of variance or Kruskal-Wallis test as appropriate. Event-free survival curves were compared using the Kaplan-Meier method. Time-dependent receiver operating characteristic (ROC) curves were used to quantify the discriminative ability of GNRI, CONUT, and PNI as continuous variables for the primary composite outcome and all-cause death.^23^ Multivariable Cox hazard regression analysis was performed to investigate the association with the primary outcome with adjustment for 24 variables (age, sex, systolic blood pressure, heart rate, New York Heart Association [NYHA] class, hypertension, diabetes mellitus, dyslipidemia, previous HF admission, AF, coronary artery disease, dementia, chronic lung disease, cancer, dialysis, angiotensin-converting enzyme inhibitor and/or angiotensin II receptor blocker use, β-blocker use, left ventricle [LV] end-diastolic volume, LV ejection fraction, severity of MR, severity of tricuspid regurgitation, and hemoglobin, estimated glomerular filtration rate, and sodium levels). Finally, we performed an additional analysis to investigate the association between the temporal change in GNRI and the primary outcome. The paired *t* test was used to test the changes in GNRI from baseline to follow-up. Statistical significance was set at *P* < 0.05. SPSS version 20 for Windows (IBM Corp, Armonk, NY) was used for the description of demographic data, survival analysis, and multivariable analysis. To perform time-dependent ROC analysis, R version 4.2.1 (R Foundation for Statistical Computing, Vienna, Austria) and “timeROC” packages were used.

## Results

**Figure 1:**
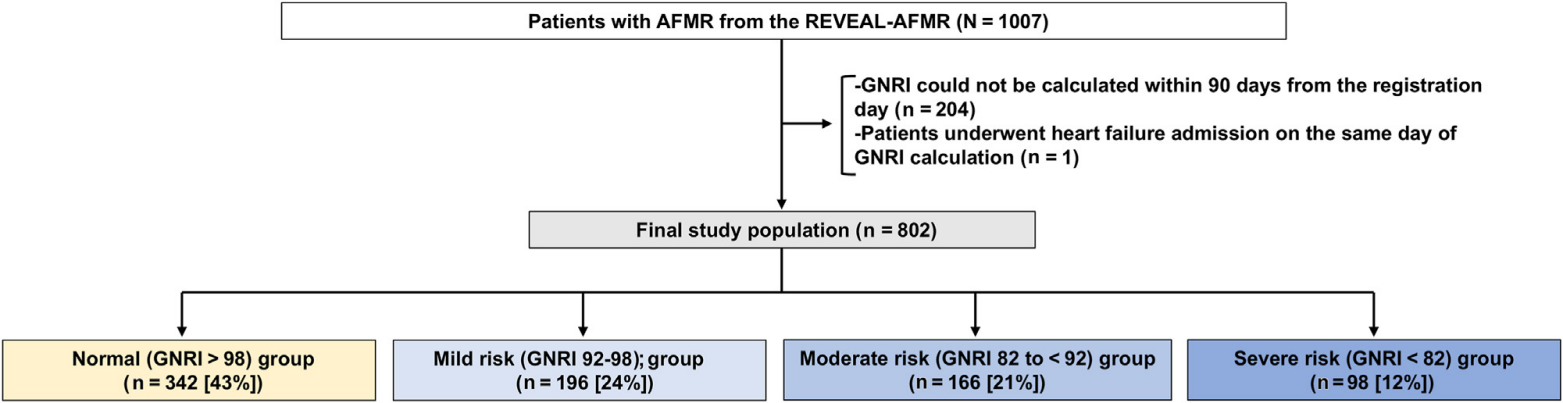
Study flow chart. The final study population was divided into 4 malnutrition risk groups according to the GNRI values (main analysis). AFMR, atrial functional mitral regurgitation; GNRI, **G**eriatric **N**utritional **R**isk **I**ndex; and REVEAL-AFMR, Real-World Observational Study for Investigating the Prevalence and Therapeutic Options for Atrial Functional Mitral Regurgitation.

Of 1007 patients with AFMR, 204 patients whose GNRI could not be obtained within 90 days from baseline were excluded. We further excluded 1 patient who developed HF exacerbation on the day of albumin measurement, leaving 802 patients for the final analysis (Fig. 1). Patients were then divided into 4 nutritional risk categories: normal (n = 342; 43%), mild risk (n = 196; 24%), moderate risk (n = 166; 21%), and severe nutritional risk (n = 98; 12%) according to their GNRI (Fig. 1). Greater nutritional risk was associated with older age, lower body mass index, a higher prevalence of NYHA functional class III or IV, previous HF hospitalization, dementia, history of cancer, and impaired activities of daily living (ADL), a shorter AF duration, lower renal function, hemoglobin levels, and cholesterol levels, and higher C-reactive protein and natriuretic peptide levels (Table 1). CONUT and PNI showed results consistent with GNRI. Echocardiographic findings showed a higher RWT, a higher prevalence of LV concentric hypertrophy, and lower stroke volume as worsening nutritional risk. There was no difference in MR severity across groups.

**Table 1:** Baseline characteristics

	All (N = 802)	Normal (n = 342)	Mild risk (n = 196)	Moderate risk (n = 166)	Severe risk (n = 98)	*P*
Age, years	78 ± 9	76 ± 9	79 ± 9	80 ± 8	80 ± 12	< 0.01
Female sex, n (%)	443 (55)	171 (50)	116 (59)	104 (63)	52 (53)	0.03
Height, cm	157 ± 10	158 ± 10	157 ± 10	155 ± 10	155 ± 10	0.01
Body weight, kg	54 ± 11	59 ± 11	54 ± 10	49 ± 11	49 ± 11	< 0.01
Body mass index, kg/m^2^	22.0 ± 3.5	23.4 ± 2.9	21.8 ± 3.2	20.1 ± 3.4	20.2 ± 3.6	< 0.01
Comorbidities						
NYHA III or IV	97 (12)	23 (7)	26 (13)	28 (17)	20 (20)	< 0.01
Previous heart failure admission	234 (29)	77 (23)	64 (33)	60 (36)	33 (34)	< 0.01
Hypertension	674 (84)	283 (83)	168 (86)	143 (86)	80 (82)	0.62
Diabetes mellitus	120 (15)	52 (15)	23 (12)	28 (17)	17 (17)	0.45
Dyslipidemia	386 (48)	185 (54)	89 (45)	69 (42)	43 (44)	0.03
Current smoker	64/781 (8)	30/328 (9)	13/192 (7)	13/164 (8)	8/97 (8)	0.82
COPD	41 (5)	16 (5)	10 (5)	10 (6)	5 (5)	0.92
Coronary artery disease	115 (14)	48 (14)	33 (17)	22 (13)	12 (12)	0.70
Dementia	64 (8)	12 (4)	13 (7)	18 (11)	21 (21)	< 0.01
Hemodialysis	24 (3)	8 (2)	5 (3)	6 (4)	5 (5)	0.43
History of cancer	172 (21)	64 (19)	41 (21)	34 (21)	33 (34)	0.02
Impaired ADL	128 (16)	19 (6)	29 (15)	43 (26)	37 (38)	< 0.01
Type of AF						0.39
Paroxysmal	108 (14)	46 (14)	25 (13)	26 (16)	11 (11)	
Persistent or permanent	542 (68)	236 (69)	139 (71)	106 (64)	61 (62)	
Sinus rhythm	152 (19)	60 (18)	32 (16)	34 (21)	26 (27)	
AF duration, months	60 (16-144) (n = 566)	72 (17-175)	84 (30-144)	52 (12-137)	36 (10-102)	0.03
Medications						
ACE inhibitor and/or ARB	363 (45)	169 (49)	88 (45)	75 (45)	31 (32)	0.02
β-Blocker	402 (50)	161 (47)	116 (59)	82 (49)	43 (44)	0.03
MRA	219 (27)	86 (25)	55 (28)	51 (31)	27 (28)	0.60
Loop diuretics	455 (57)	182 (53)	112 (57)	104 (63)	57 (58)	0.24
Anticoagulant	576 (72)	258 (75)	153 (78)	109 (66)	56 (57)	< 0.01
Laboratory data						
White blood cells, per μL	5718 ± 2377 (n = 800)	5461 ± 1909	5333 ± 1802	5811 ± 2467	7225 ± 3756	< 0.01
Lymphocyte count, per μL	1304 ± 624 (n = 590)	1448 ± 648	1285 ± 565	1142 ± 593	1109 ± 567	< 0.01
Hemoglobin, g/dL	11.7 ± 2.0 (n = 800)	12.6 ± 1.8	11.7 ± 1.8	11.1 ± 1.9	9.8 ± 1.9	< 0.01
Albumin, g/dL	3.7 ± 0.6	4.2 ± 0.2	3.8 ± 0.2	3.5 ± 0.3	2.6 ± 0.5	< 0.01
Total bilirubin, mg/dL	0.9 ± 0.7 (n = 745)	0.9 ± 0.4	0.9 ± 0.5	1.0 ± 1.3	0.7 ± 0.4	0.08
Blood urea nitrogen, mg/dL	24 ± 14 (n = 794)	22 ± 11	24 ± 14	25 ± 13	29 ± 20	< 0.01
Creatinine, mg/dL	1.3 ± 1.2 (n = 801)	1.1 ± 0.9	1.4 ± 1.3	1.3 ± 1.2	1.6 ± 1.4	0.01
Sodium, mEq/L	140 ± 3 (n = 796)	140 ± 3	140 ± 3	140 ± 3	139 ± 4	< 0.01
Total cholesterol, mg/dL	169 ± 39 (n = 534)	179 ± 35	163 ± 34	165 ± 42	138 ± 39	< 0.01
C-reactive protein, mg/dL	0.1 (0.1-0.6) (n = 701)	0.1 (0.0-0.2)	0.1 (0.1-0.4)	0.3 (0.1-1.1)	2.2 (0.8-7.0)	< 0.01
BNP, pg/mL	208 (117-403) (n = 441)	147 (89-256)	260 (125-470)	260 (140-442)	425 (257-707)	< 0.01
NT-proBNP, pg/mL	1378 (629-3017) (n = 278)	955 (426-1550)	1738 (722-3823)	1770 (1016-4349)	3543 (1907-4934)	< 0.01
GNRI	94.3 ± 10.0	102.6 ± 3.6	94.9 ± 1.7	88.0 ± 2.6	74.6 ± 7.5	< 0.01
CONUT	3 (1-4) (n = 419)	2 (1-3)	3 (2-4)	4 (2-5)	8 (6-9)	< 0.01
PNI	43.6 ± 0.3 (n = 590)	48.9 ± 4.0	43.9 ± 3.7	40.3 ± 4.5	31.0 ± 5.5	< 0.01
Vital signs						
Systolic blood pressure, mm Hg	127 ± 20 (n = 760)	128 ± 19	126 ± 20	125 ± 23	125 ± 20	0.46
Diastolic blood pressure, mm Hg	70 ± 14 (n = 760)	71 ± 13	69 ± 15	70 ± 14	67 ± 15	0.07
Heart rate, bpm	73 ± 17 (n = 794)	72 ± 16	73 ± 16	73 ± 17	79 ± 20	< 0.01
Echocardiographic values						
IVSd, mm	10.0 ± 1.9	10.0 ± 1.9	9.7 ± 1.8	9.9 ± 2.1	10.5 ± 2.1	0.02
LVDd, mm	49 ± 7	49 ± 7	49 ± 7	48 ± 7	47 ± 7	< 0.01
LVDs, mm	32 ± 6	32 ± 6	32 ± 6	32 ± 6	31 ± 6	0.45
LVEDV, mL	97 ± 36	99 ± 37	96 ± 35	96 ± 36	92 ± 37	0.37
LVESV, mL	37 ± 16	38 ± 16	37 ± 15	37 ± 15	37 ± 18	0.87
Relative wall thickness	0.41 ± 0.10	0.40 ± 0.09	0.40 ± 0.09	0.42 ± 0.10	0.45 ± 0.11	< 0.01
LV mass index, g/m^2^	115 ± 33	113 ± 31	114 ± 33	117 ± 34	121 ± 34	0.15
LV mass/LVEDV, g/mL	2.0 ± 0.7	2.0 ± 0.7	1.9 ± 0.7	1.9 ± 0.8	2.1 ± 0.8	0.42
Concentric hypertrophy	220 (27)	73 (21)	55 (28)	55 (33)	37 (38)	< 0.01
LVEF, %	62 ± 6	62 ± 6	62 ± 6	61 ± 6	61 ± 7	0.31
LV stroke volume, mL	59 ± 21 (n = 603)	62 ± 21	59 ± 22	55 ± 21	54 ± 21	0.01
Cardiac index, L/min/m^2^	2.6 ± 1.0 (n = 602)	2.6 ± 0.9	2.7 ± 1.0	2.7 ± 1.0	2.6 ± 1.0	0.51
LA diameter, mm	51 ± 11 (n = 800)	52 ± 11	52 ± 10	51 ± 11	48 ± 9	< 0.01
LAVI, mL/m^2^	95 ± 57 (n = 749)	95 ± 59	96 ± 53	99 ± 62	87 ± 49	0.48
TR velocity (m/s)	2.8 ± 0.5 (n = 772)	2.7 ± 0.5	2.8 ± 0.4	2.9 ± 0.4	2.8 ± 0.4	< 0.01
PASP, mm Hg	39 ± 12 (n = 670)	37 ± 13	41 ± 12	41 ± 12	40 ± 11	< 0.01
IVC maximum, mm	18 ± 6 (n = 791)	17 ± 6	18 ± 6	18 ± 6	17 ± 6	0.21
IVC minimum, mm	9 ± 6 (n = 699)	9 ± 5	9 ± 6	10 ± 6	9 ± 6	0.21
TAPSE, mm	18 ± 4 (n = 372)	18 ± 4	17 ± 4	18 ± 4	17 ± 5	0.28
MR ERO, cm^2^	0.26 ± 0.11 (n = 361)	0.27 ± 0.12	0.27 ± 0.12	0.26 ± 0.12	0.24 ± 0.09	0.55
MR RVol, mL	45 ± 19 (n = 444)	45 ± 18	44 ± 16	46 ± 24	43 ± 21	0.78
Mitral E wave, cm/s	102 ± 28 (n = 792)	101 ± 28	104 ± 27	103 ± 30	103 ± 25	0.53
e’ (sep), cm/s	7.0 ± 2.5 (n = 747)	7.1 ± 2.3	7.1 ± 2.7	7.0 ± 2.9	6.5 ± 2.5	0.19
E/e’ (sep)	16.2 ± 7.1 (n = 742)	15.5 ± 7.0	16.3 ± 7.1	16.6 ± 7.2	17.6 ± 7.3	0.07
Deceleration time, ms	183 ± 54 (n = 787)	187 ± 53	180 ± 54	181 ± 49	183 ± 54	0.46
Severe AS	45 (6)	17 (5)	10 (5)	8 (5)	10 (10)	0.25
Severe AR	10 (1)	2 (1)	3 (2)	4 (2)	1 (1)	0.30
Severe TR	128 (16)	42 (12)	34 (17)	37 (22)	15 (15)	0.03
MR grades						0.44
Moderate	563 (70)	244 (71)	134 (68)	112 (68)	73 (75)	
Moderate to severe	125 (16)	55 (16)	35 (18)	22 (13)	13 (13)	
Severe	114 (14)	43 (13)	27 (14)	32 (19)	12 (12)	
Treatment for AFMR						
Mitral valve surgery	95 (12)	48 (14)	23 (12)	19 (11)	5 (5)	0.10
MitraClip	27 (3)	7 (2)	9 (5)	10 (6)	1 (1)	0.05

Data are expressed as a mean ± SD, median (interquartile range), or n (%). Normally distributed continuous variables were compared using 1-way analysis of variance. Otherwise, continuous variables were compared using a Kruskal-Wallis test. Fisher exact test was used to analyze categorical variables. MitraClip is from Abbott Laboratories (Chicago, IL).

ACE, angiotensin-converting enzyme; ADL, activity of daily living; AF, atrial fibrillation; AFMR, atrial functional mitral regurgitation; AR, aortic regurgitation; ARB, angiotensin II receptor blocker; AS, aortic stenosis; BNP, brain natriuretic peptide; bpm, beats per minute; CONUT, Controlling Nutritional Status; COPD, chronic obstructive pulmonary disease; e’, early diastolic tissue velocity; E/e’, early mitral inflow velocity divided by mitral annular early diastolic velocity; ERO, effective regurgitant orifice; GNRI, **G**eriatric **N**utritional **R**isk **I**ndex; IVC, inferior vena cava; IVSd, intraventricular septal thickness at diastole; LA, left atrium; LAVI, left atrial volume index; LV, left ventricle; LVDd, left ventricular end-diastolic diameter; LVDs, left ventricular end-systolic diameter; LVEDV, left ventricular end-diastolic volume; LVEF, left ventricular ejection fraction; LVESV, left ventricular end-systolic volume; MR, mitral regurgitation; MRA, mineralocorticoid receptor antagonist; NT-proBNP, N-terminal pro hormone brain natriuretic peptide; NYHA, New York Heart Association; PASP, pulmonary artery systolic pressure; PNI, **P**rognostic **N**utritional **I**ndex; RVol, regurgitant volume; sep, septal; TAPSE, tricuspid annular plane systolic excursion; TR, tricuspid regurgitation.

### Outcome analysis

**Figure 2:**
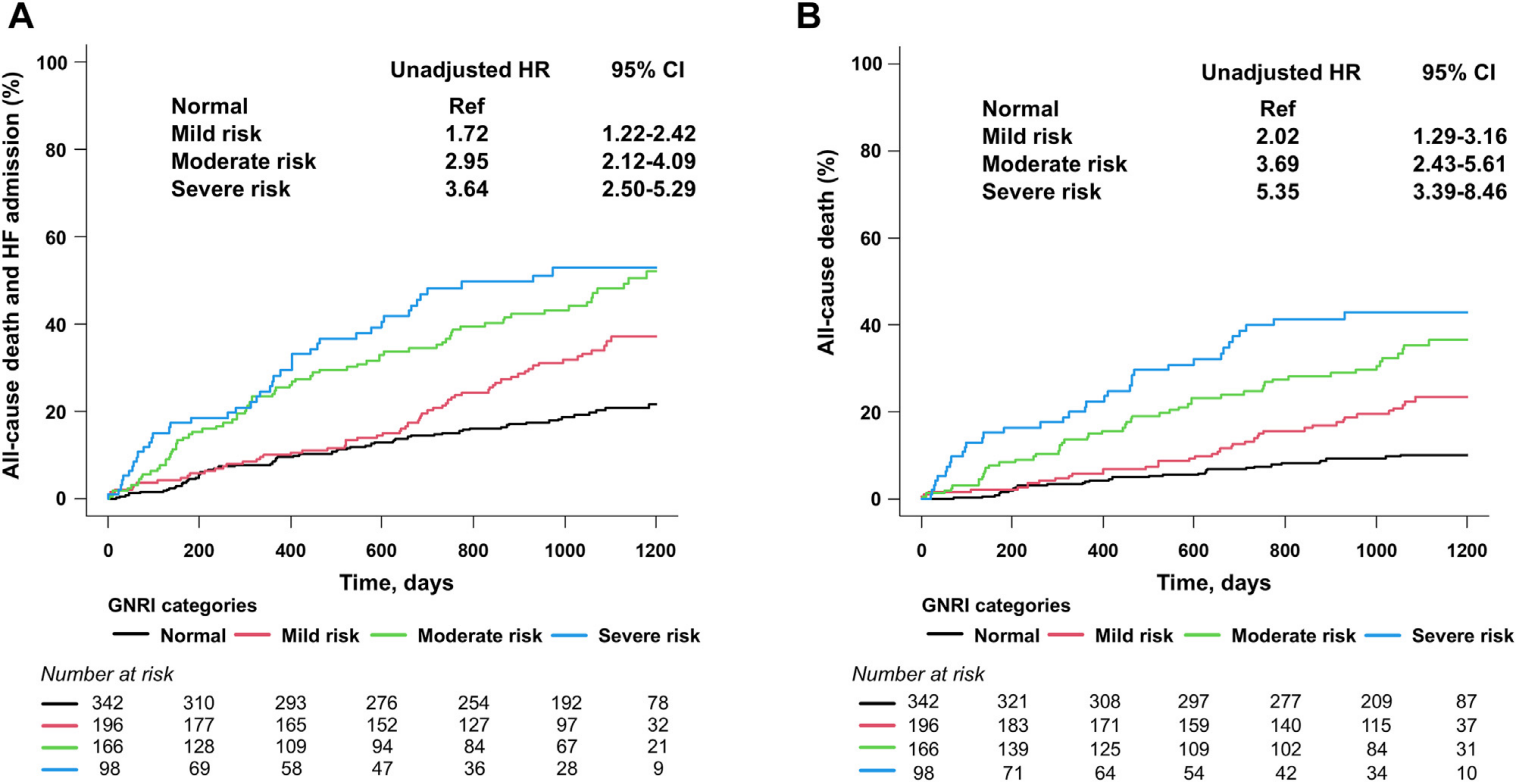
Kaplan-Meier curve analysis for the primary outcome and all-cause death. The severity of malnutrition risk was associated with higher rates of the composite outcome of all-cause mortality and heart failure (HF) admission (**A**) and all-cause mortality (**B**). GNRI, **G**eriatric **N**utritional **R**isk **I**ndex; CI, confidence interval; HR, hazard ratio; Ref, reference.

During the median follow-up of 978 (IQR, 492-1141) days, 254 primary composite outcomes and 169 all-cause deaths were observed, with HF and cancer being the 2 most common causes of death (Table 2). Kaplan-Meier curves and univariable Cox hazard models showed that the rate of composite outcome of all-cause mortality and HF hospitalization was the highest in the severe malnutritional risk group, followed by the moderate risk group, mild risk group, and normal group (log-rank *P* < 0.01; Fig. 2A). The corresponding event rates of all-cause death were increased as the severity of malnutrition risk increased (log-rank *P* < 0.01; Fig. 2B). Sensitivity analyses performed separately among patients with and without AF (n = 650 and 152, respectively) showed similar associations between the GNRI risk categories and the primary outcome, which suggests that the presence of AF did not significantly influence the results (Supplemental Fig. S1).

**Table 2:** Details of deaths

	All (N = 802)
CV death	82 (10)
Non-CV death	87 (11)
Details of deaths	
CV deaths	
Heart failure	38 (5)
Cerebral infarction	9 (1)
Sudden death	16 (2)
Other CV death	19 (2)
Non-CV deaths	
Infection	23 (3)
Cancer	37 (5)
Other non-CV death	27 (3)

Data are expressed as a n (%).

CV, cardiovascular.

Increasing severity of malnutrition risk categories remained significantly associated with the primary outcome after adjusting for 24 confounding factors (normal risk group as a reference): mild risk (adjusted hazard ratio [HR], 1.52; 95% confidence interval [CI], 1.04-2.22; *P* = 0.03), moderate risk (adjusted HR, 2.58; 95% CI, 1.77-3.74; *P* < 0.01), and severe risk (adjusted HR, 2.44; 95% CI, 1.53-3.89; *P* < 0.01). Similarly, the severity of malnutrition risk was associated with all-cause mortality even after adjusting for the 24 confounders (normal group as a reference): mild risk (adjusted HR, 1.74; 95% CI, 1.06-2.87; *P* = 0.03), moderate risk (adjusted HR, 3.74; 95% CI, 2.32-6.03; *P* < 0.01), and severe risk (adjusted HR, 3.47; 95% CI, 1.93-6.24; *P* < 0.01).

When analyzing GNRI as a continuous variable (per 1 standard deviation [1SD; = 10 points] increment), it was associated with the primary composite outcome (HR, 0.68; 95% CI, 0.61-0.76; *P* < 0.01) and all-cause death (HR, 0.62; 95% CI, 0.54-0.70; *P* < 0.01). Even after adjusting for the 24 confounding factors, GNRI (per 1SD increment) remained an independent predictor of the primary outcome (adjusted HR, 0.76 per 1SD increment; 95% CI, 0.66-0.87; *P* < 0.01) and all-cause death (adjusted HR, 0.69 per 1SD increment; 95% CI, 0.58-0.82; *P* < 0.01). There was no interaction of MR severity (moderate or severe) on the association between GNRI and outcomes (*P* interaction = 0.33 for the primary outcome and 0.45 for all-cause death).

When analyzing cardiovascular (CV) and non-CV death separately, GNRI was associated with both outcomes, with a numerically greater effect on non-CV deaths (HR, 0.71 per 1SD increment; 95% CI, 0.58-0.87; *P* < 0.01 for CV death; and HR, 0.55 per 1SD increment; 95% CI, 0.46-0.65; *P* < 0.01 for non-CV death).

We confirmed that the severity of malnutrition risk assessed using CONUT and PNI were associated with the composite outcome and all-cause mortality in patients with AFMR (Supplemental Figs. S2 and S3). The time-dependent ROC analysis revealed that GNRI, CONUT, and PNI as continuous variables exhibited modest predictive accuracy for the primary outcome or all-cause mortality (approximately 0.70) with no statistical difference among the 3 tools at 1, 2, and 3 years (Supplemental Table S1).

### Temporal change in GNRI and outcomes

**Figure 3:**
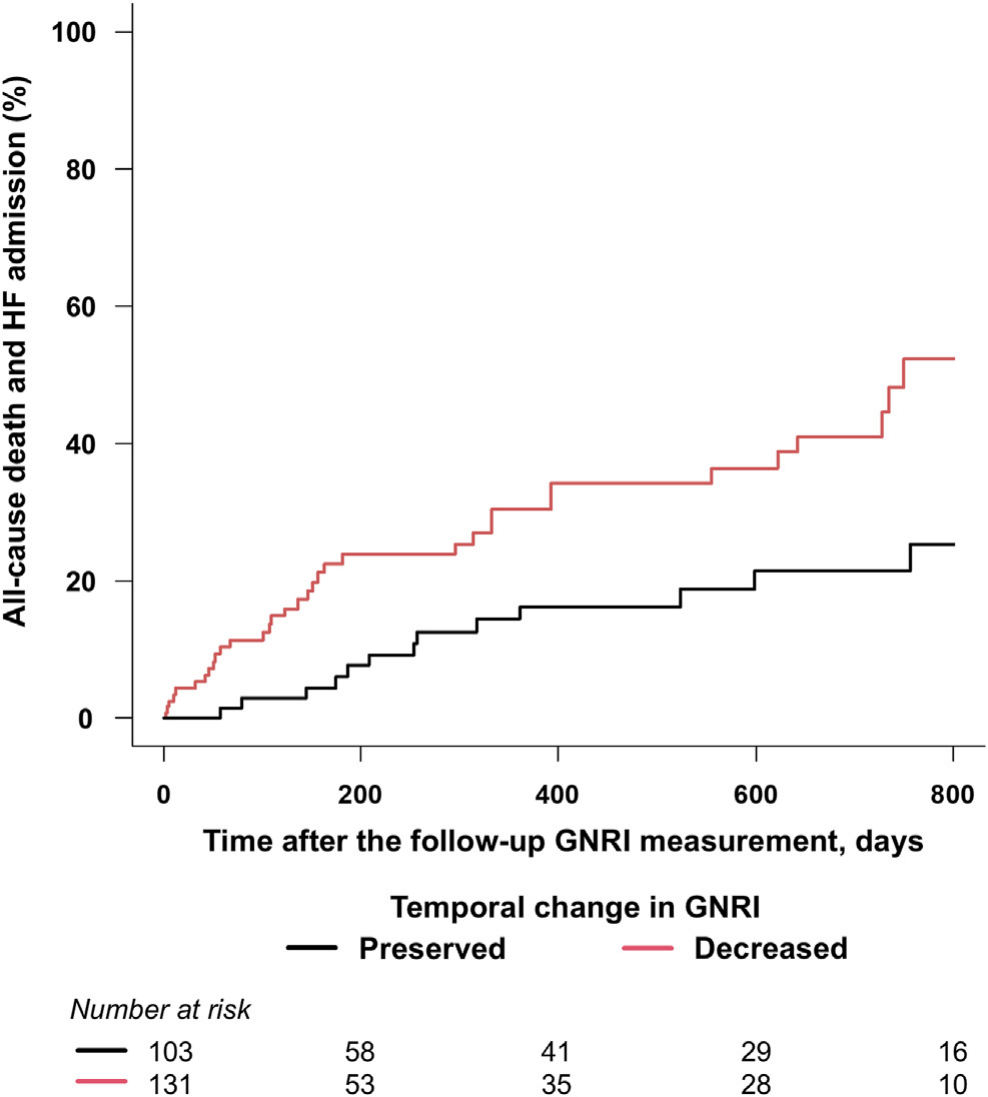
Kaplan-Meier curve analysis for the association between temporal changes in **G**eriatric **N**utritional **R**isk **I**ndex (GNRI) and the primary outcome. Time zero was set as the date of the follow-up GNRI measurement. Patients with a decreased GNRI over time had a higher incidence of the primary outcome than those with a preserved GNRI. HF, heart failure.

To investigate the prognostic significance of temporal change in GNRI, 234 patients with follow-up GNRI data (29.2%) were examined. The patients with follow-up GNRI showed a lower prevalence of cancer and impaired ADL, more frequent valve surgery for MV, and greater hemoglobin levels and nutritional status than those without (Supplemental Table S2). The median time between baseline and follow-up was 683 (IQR, 260-988) days. Overall, mean GNRI values were significantly decreased from baseline to follow-up (95.8 ± 8.8 to 93.9 ± 10.9; *P* = 0.01). After the follow-up GNRI measurement, 51 primary outcomes were observed during 178 (IQR, 31-592) days. Patients with a decreased GNRI value had a higher incidence of the primary outcome (27%) than those with a preserved GNRI (15%; log-rank, *P* < 0.01; Fig. 3). A decreased GNRI value was associated with higher rates of the primary outcome compared with a preserved GNRI value in a univariable model (unadjusted HR, 2.53; 95% CI, 1.37-4.66; *P* < 0.01) and a multivariable model adjusted for age, sex, baseline GNRI, and time between baseline and follow-up (adjusted HR, 3.83; 95% CI, 1.97-7.43; *P* < 0.01).

## Discussion

To the best of our knowledge, this was the first large-scale study to characterize the prevalence, clinical features, and outcomes of malnutrition risk in patients with AFMR (Central Illustration). We showed that: (1) at least mild malnutrition risk assessed using the GNRI was present in 57% of patients with AFMR; (2) worsening nutritional status was associated with more vulnerable conditions, including older age, lower body mass index, lower renal function, more anemia, and a higher prevalence of dementia and impaired ADL; (3) the severity of malnutrition risk was associated with a higher risk of the primary composite outcome after adjusting for multiple confounding factors; and (4) temporal worsening in the GNRI showed an independent association with the composite outcome. These data emphasize the importance of using the GNRI for assessing malnutrition risk and identifying vulnerable populations in patients with AFMR.

**Central Illustration:**
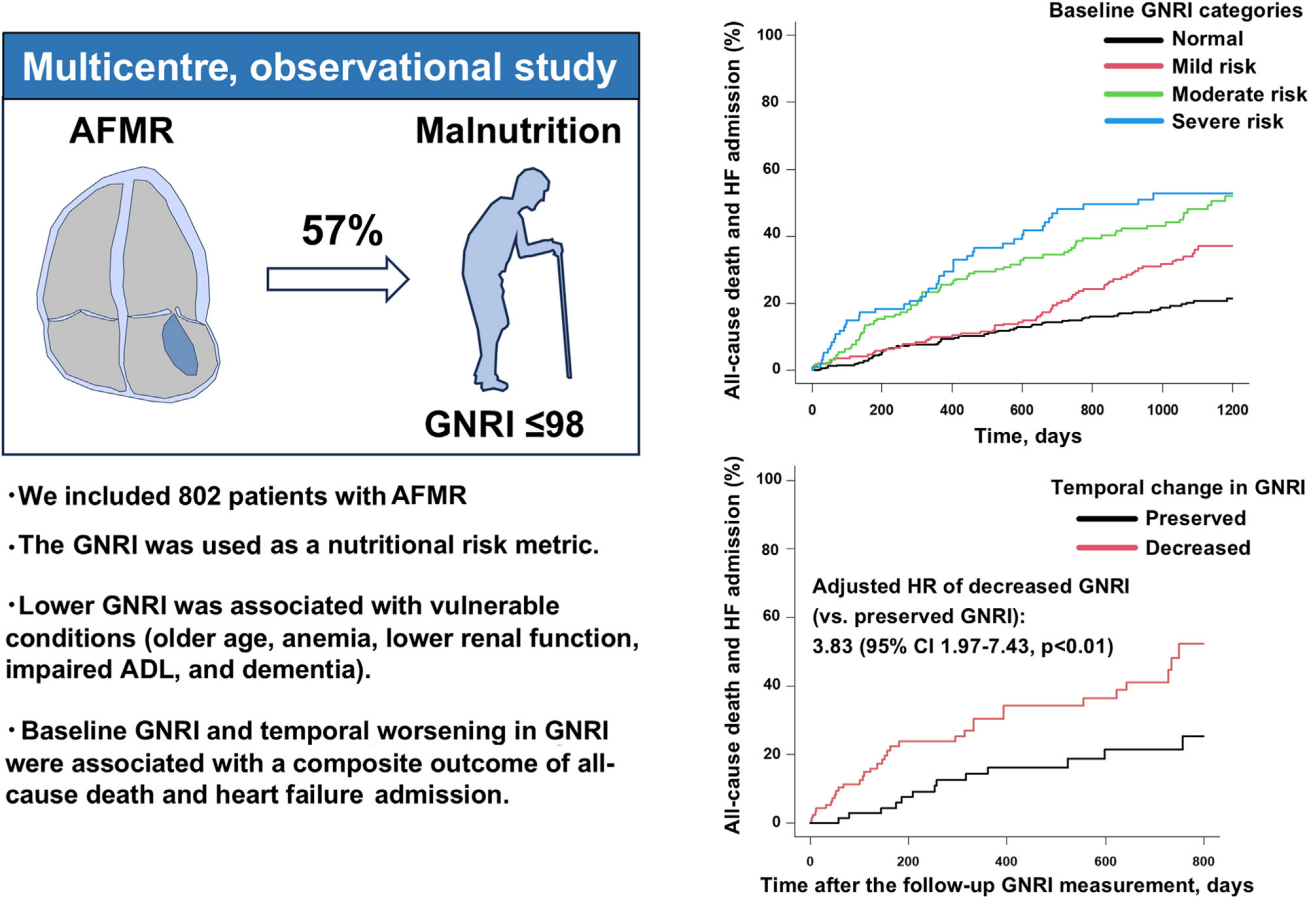
Characteristics of patients with atrial functional mitral regurgitation and malnutrition. Patients with atrial functional mitral regurgitation (AFMR) and malnutrition represent a vulnerable population with worse clinical outcomes. ADL, activities of daily living; GNRI, **G**eriatric **N**utritional **R**isk **I**ndex; HF, heart failure.

### Prevalence of malnutrition in patients with AFMR

In the current study, at least mild nutrition risk (GNRI ≤ 98) was common in patients with AFMR (57%). A large number of studies so far investigated the prevalence of malnutrition in patients with various CV diseases, and results vary widely possibly because of the study populations, different nutritional assessment tools, or cutoffs used.^8^, ^9^, ^10^, ^11^, ^12^, ^13^, ^14^, ^15^ For example, a substudy of the **T**reatment **O**f **P**reserved **C**ardiac Function Heart Failure with an **A**ldosterone An**T**agonist (TOPCAT) trial revealed that 37% of patients with HFpEF, with a median age of 72 years old, had at least mild nutrition risk (GNRI ≤ 98).^8^ A Japanese multicentre study, involving 890 hospitalized patients with HF aged 65 years and older, reported that 42% of patients had malnutrition assessed using the Global Leadership Initiative on Malnutrition (GLIM) criteria.^9^ Age is a major driver of malnutrition.^9^^,^^10^^,^^12^^,^^14^ Although we cannot rule out the possibility that the numerically higher prevalence of malnutrition in the current study than in other studies could be related to older age (mean age, 78 years old in ours), it is important to recognize that more than half of the cases with AFMR were at nutritional risk.

### Characterization of malnutrition in patients with AFMR

We observed that malnourished patients with AFMR had a higher prevalence of NYHA III or IV class and higher natriuretic peptide levels, which is in agreement with previous studies on other diseases.^8^^,^^10^^,^^13^^,^^14^^,^^24^ These data suggest that concomitant HF plays a key role in developing malnutrition in patients with AFMR. HF can trigger a complex interplay of neurohormonal derangement, inflammation, anabolic-catabolic imbalance, appetite suppression, malabsorption, and reduced physical activity, contributing to the development of malnutrition and muscle wasting.^25^^,^^26^

Increased C-reactive protein levels, lower renal function, anemia, and impaired ADL observed in patients with lower GNRI categories might be a result of this vicious cycle. Minamisawa et al. reported that hemoglobin and renal function decreased in patients with HFpEF and worse GNRI values. (8) In addition, Maeda et al. reported a noteworthy overlap among frailty, sarcopenia, cachexia, and malnutrition in older adults with HF.^27^ Another study reported the association between cognitive impairment, poor ADL, and malnutrition in outpatients with cardiometabolic disease.^28^ Collectively, our and other data suggest that malnutrition represents a marker for patients with vulnerability in patients with AFMR.

### Prognostic effect of malnutrition in patients with AFMR

In this study, we first showed the association between malnutrition risk assessed using the GNRI and clinical outcomes in patients with AFMR. The association between malnutrition and outcomes was confirmed using other nutritional risk assessment tools (CONUT and PNI). In addition to a multicentre design, an adequate number of well known prognostic factors were adjusted in the multivariable analysis. These might assure the generalizability and robustness of our findings.

We revealed that GNRI risk categories and a continuous metric of GNRI values were associated with worse clinical outcomes. Because risk category is more easily interpreted, it will be useful for estimating the risk of patients with AFMR in daily practice. In addition, our results indicate that patients with a lower GNRI value have poorer prognosis even compared with those in the same GNRI category.

It is noteworthy that the severity of malnutrition risk was associated with CV and non-CV deaths, particularly with the latter. Malnutrition is often accompanied by multiple comorbidities as in the current study.^10^ The current data suggest that, in addition to dietary intervention, the treatment of comorbidities is critical in the management of malnutrition in patients with AFMR. In this study, we could not find the superiority of the GNRI compared with the CONUT and PNI in predicting the poor prognosis of patients with AFMR. However, unlike CONUT and PNI, GNRI is a parameter that combines anthropometric metrics (the ratio of body weight to ideal body weight) and a serum biomarker (albumin levels). This might allow for assessment of coexisting physical frailty in addition to nutritional status.

Of note, nutritional status changes over time.^15^^,^^29^ We showed that temporal worsening in GNRI value was independently associated with poor prognosis irrespective of baseline GNRI value. Gonzalez Ferreiro et al. reported that nearly 70% of patients with aortic stenosis and nutritional risk (GNRI ≤ 98) showed an improvement in nutritional status (GNRI > 98) 3 months after transcatheter aortic valve replacement, and residual nutritional risk after transcatheter aortic valve replacement was associated with poor prognosis.^29^ Although we cannot determine the causality whether improved nutritional status led to better outcomes or whether improved disease status improved nutritional status, these data and ours support the importance of nutrition status in the outcome of AFMR patients.

### Clinical implications

Malnutrition was associated with vulnerable conditions in patients with AFMR, including HF severity, anemia, reduced renal function, impaired ADL, and dementia. Because the GNRI is simple, and can be used promptly, and repeatedly, it might be a good screening tool for detecting vulnerable patients who require comprehensive care. In cases of coexisting HF, optimization of guideline-directed medical therapy is essential. In addition, surgical intervention for AFMR and/or ablation for AF might improve HF.^1^^,^^4^ Furthermore, support for coexisting physical frailty and dementia,^30^ as well as individualized nutritional treatment,^31^ might contribute to a favourable outcome in malnourished patients with AFMR.

### Limitations

This study had the following limitations. Despite the multicentre design, all participants were Japanese. The diagnosis of AFMR was on the basis of TTE results. However, all TTE images of patients with AFMR were carefully reviewed by experienced cardiologists at each institution. Furthermore, the agreement in the diagnosis of AFMR was confirmed in randomly selected patients. Echocardiographic measurements were performed at each institution, but the measurements were confirmed at the core laboratory, and these parameters showed excellent agreement with the original measurements at each site.^4^ There might be some differences between body weight and albumin measurements. Furthermore, serum albumin is not a specific marker of nutritional status because it cannot reliably distinguish nutritional risk from systemic illness or inflammation. Body weight might be insensitive to critical changes in body composition. Therefore, GNRI calculated from serum albumin levels and body weight might not accurately reflect nutritional status in some patients with systemic disease, inflammation, or volume retention. Although we believe that the GNRI is a valuable tool for estimating risk in older adults with AFMR, the GLIM criteria, which incorporate more comprehensive elements (body composition changes and inflammation), might be more reliable for diagnosing malnutrition. Because the REVEAL-AFMR registry included patients with moderate or greater MR, data were not available for patients with less than moderate MR. Therefore, we could not fully investigate the effect of the entire spectrum of MR severity on the association between GNRI and prognosis. Of 802 patients, only 234 patients could be used in the additional analysis on follow-up GNRI, resulting in selection bias. Patients with follow-up GNRI showed better baseline nutritional status and less vulnerable conditions than those without. Thus, the effect of follow-up GNRI might be underestimated. Further, confounding factors could not be adjusted adequately because of the small number of events in the additional analysis. Caution should be paid in the interpretation. Nevertheless, the evidence of temporal change in nutritional status remains sparse.^15^^,^^29^

### Conclusions

Malnutrition assessed using the GNRI was common and was associated with multiple comorbid conditions and poor clinical outcomes in patients with AFMR. These data emphasize the importance of the GNRI for the assessment of malnutrition risk in patients with AFMR and suggest that those with malnutrition risk represent vulnerable populations with a high risk of worse outcomes.
