# Supplementary material for: Characterization of Malnutrition in Atrial Functional Mitral Regurgitation
Source: CJC Open. 2025 May 21;7(8):1038–47. doi: 10.1016/j.cjco.2025.05.007 (PMC12399123; doi:10.1016/j.cjco.2025.05.007)
Supplement: Supplementary Material [file mmc1.docx]

**SUPPLEMENTARY MATERIALS for: Characterization of Malnutrition in Atrial Functional Mitral Regurgitation**

Tsukasa Murakami, MD, PhD^1,2^, Nobuyuki Kagiyama, MD, PhD^3^, Tomohiro Kaneko, MD, PhD^3^, Kazuki Kagami, MD, PhD^1^, Masashi Amano, MD, PhD^4^, Taiji Okada, MD, PhD^5^, Yukio Sato, MD, PhD^6^, Yohei Ohno, MD, PhD^7^, Kimi Sato, MD, PhD^8^, Kojiro Morita, MPH, PhD, RN, PHN^9^, Tomoko Machino-Ohtsuka, MD, PhD^8^, Yukio Abe, MD, PhD^10^, Hideki Ishii, MD, PhD^1^, Masaru Obokata, MD, PhD^1^

**Address for correspondence**:

Masaru Obokata, MD, PhD

Department of Cardiovascular Medicine

Gunma University Graduate School of Medicine,

3-39-22 Showa-machi, Maebashi, Gunma 371-8511, Japan

Tel.: +81-27-220-8145; Fax: +81-27-220-8158

E-mail address: [obokata.masaru@gunma-u.ac.jp](mailto:obokata.masaru@gunma-u.ac.jp)

**Supplemental Tables:**

**Supplemental Table S1. Time-dependent ROC analysis for each nutritional assessment tool**

|  | **AUC at a given time point** | | | **P-values** | | |
| --- | --- | --- | --- | --- | --- | --- |
|  | GNRI (N=802) | CONUT (N=419) | PNI  (N=590) | GNRI vs CONUT (N=419) | GNRI vs PNI (N=590) | CONUT vs PNI (N=419) |
| *HF admission and all-cause death* |  |  |  |  |  |  |
| 1 year | 0.67 | 0.70 | 0.69 | 0.54 | 0.62 | 0.70 |
| 2 years | 0.68 | 0.67 | 0.69 | 0.75 | 0.47 | 0.80 |
| 3 years | 0.67 | 0.69 | 0.66 | 0.17 | 0.82 | 0.50 |
| *All-cause death* |  |  |  |  |  |  |
| 1 year | 0.71 | 0.71 | 0.70 | 0.53 | 0.83 | 0.73 |
| 2 years | 0.72 | 0.67 | 0.70 | 0.61 | 0.77 | 0.74 |
| 3 years | 0.70 | 0.68 | 0.68 | 0.49 | 0.86 | 0.64 |

Abbreviations: AUC, Area Under the Curve; CONUT, Controlling Nutritional Status; GNRI, Geriatric Nutritional Risk Index; HF, heart failure; PNI, Prognostic nutritional index; ROC, Receiver Operating Characteristic.

**Supplemental Table S2. Differences in characteristics between patients with follow-up GNRI data and those without**

|  | Patients without follow-up GNRI (N=568) | Patients with follow-up GNRI (N=234) | P-value |
| --- | --- | --- | --- |
| Age (years) | 78±9 | 77±10 | 0.06 |
| Female, n (%) | 311 (55) | 132 (56) | 0.70 |
| Height (cm) | 157±10 | 157±10 | 0.53 |
| Body weight (kg) | 54±11 | 55±12 | 0.23 |
| Body mass index (kg/m^2^) | 21.9±3.4 | 22.2±3.7 | 0.25 |
| ***Comorbidities*** |  |  |  |
| NYHA, n (%) |  |  | 0.53 |
| I or II | 504 (89) | 201 (86) |  |
| III | 51 (9) | 27 (12) |  |
| IV | 13 (2) | 6 (3) |  |
| NYHA III or IV, n (%) | 64 (11) | 33 (14) | 0.28 |
| Prior heart failure admission, n (%) | 161 (28) | 73 (31) | 0.44 |
| Hypertension, n (%) | 476 (84) | 198 (85) | 0.83 |
| Diabetes mellitus, n (%) | 87 (15) | 33 (14) | 0.74 |
| Dyslipidemia, n (%) | 272 (48) | 114 (49) | 0.88 |
| Current smoker, n (%) | 40/554 (7) | 24/227 (11) | 0.15 |
| COPD, n (%) | 32 (6) | 9 (4) | 0.38 |
| Coronary artery disease, n (%) | 74 (13) | 41 (18) | 0.12 |
| Dementia, n (%) | 51 (9) | 13 (6) | 0.12 |
| Hemodialysis, n (%) | 16 (3) | 8 (3) | 0.65 |
| History of cancer, n (%) | 141 (25) | 31 (13) | <0.01 |
| Impaired ADL, n (%) | 106 (19) | 22 (9) | <0.01 |
| Type of AF, n (%) |  |  | 0.10 |
| Paroxysmal | 85 (15) | 23 (10) |  |
| Persistent or permanent | 382 (67) | 160 (68) |  |
| Sinus rhythm | 101 (18) | 51 (22) |  |
| AF duration (months) | 60 (18-144) | 72 (12-156) | 0.50 |
| ***Medications*** |  |  |  |
| ACE-inhibitor, ARB, n (%) | 242 (43) | 121 (52) | 0.02 |
| β-blocker, n (%) | 282 (50) | 120 (51) | 0.70 |
| MRA, n (%) | 146 (26) | 73 (31) | 0.12 |
| Loop diuretics, n (%) | 324 (57) | 131 (56) | 0.81 |
| Anti-coagulant, n (%) | 408 (72) | 168 (72) | 1.00 |
| ***Laboratory data*** |  |  |  |
| White blood cell (/µL) | 5789±2500 | 5545±2045 | 0.19 |
| Lymphocyte count (/µL) | 1283±599 | 1354±677 | 0.21 |
| Hemoglobin (g/dL) | 11.6±2.1 | 12.0±1.9 | 0.02 |
| Albumin (g/dL) | 3.7±0.6 | 3.8±0.5 | 0.01 |
| Total bilirubin (mg/dL) | 0.9±0.8 | 0.9±0.4 | 0.64 |
| Blood urea nitrogen (mg/dL) | 25±14 | 23±13 | 0.10 |
| Creatinine (mg/dL) | 1.3±1.2 | 1.2±1.1 | 0.60 |
| Sodium (mEq/L) | 140±3 | 140±3 | 0.96 |
| Total cholesterol (mg/dL) | 168±40 | 169±35 | 0.70 |
| C-reactive protein (mg/dL) | 0.2 (0.1-0.9) | 0.1 (0.0-0.4) | 0.03 |
| BNP (pg/mL) | 208 (113-381) | 210 (121-431) | 0.54 |
| NT-proBNP (pg/mL) | 1447 (669-3326) | 1358 (603-2622) | 0.62 |
| GNRI | 93.7±10.4 | 95.8±8.8 | 0.01 |
| CONUT | 3 (1-4) | 2 (1-4) | 0.03 |
| PNI | 43.0±7.6 | 45.1±6.5 | <0.01 |
| ***Vital signs*** |  |  |  |
| Systolic blood pressure (mmHg) | 127±20 | 126±20 | 0.48 |
| Diastolic blood pressure (mmHg) | 70±14 | 70±13 | 0.64 |
| Heart rate (bpm) | 73±18 | 73±15 | 0.54 |
| ***Echocardiographic values*** |  |  |  |
| IVSd (mm) | 10±2 | 10±2 | 0.24 |
| LVDd (mm) | 49±7 | 49±7 | 0.59 |
| LVDs (mm) | 32±6 | 32±6 | 0.78 |
| LVEDV (mL) | 96±36 | 99±36 | 0.34 |
| LVESV (mL) | 37±16 | 38±16 | 0.43 |
| Relative wall thickness | 0.42±0.09 | 0.40±0.10 | 0.10 |
| LV mass index (g/m²) | 116±32 | 112±33 | 0.17 |
| LV mass/LVEDV (g/mL) | 2.0±0.7 | 1.9±0.7 | 0.05 |
| Concentric hypertrophy, n (%) | 165 (29) | 55 (24) | 0.12 |
| LVEF (%) | 62±6 | 62±6 | 0.48 |
| LV stroke volume (mL) | 59±22 | 58±21 | 0.51 |
| Cardiac index (L/min/m²) | 2.6±0.9 | 2.6±1.0 | 0.82 |
| LA diameter (mm) | 51±11 | 52±10 | 0.73 |
| LAVI (mL/m²) | 93±57 | 98±56 | 0.30 |
| TR velocity (m/s) | 2.8±0.5 | 2.8±0.5 | 0.17 |
| PASP (mmHg) | 38±12 | 40±13 | 0.07 |
| IVC max (mm) | 17±6 | 18±6 | 0.13 |
| IVC min (mm) | 9±5 | 9±6 | 0.43 |
| TAPSE (mm) | 18±4 | 18±4 | 0.38 |
| MR ERO (cm²) | 0.26±0.12 | 0.27±0.11 | 0.65 |
| MR RVol (mL) | 45±20 | 44±16 | 0.33 |
| Mitral E wave (cm/s) | 102±27 | 104±29 | 0.33 |
| e’ (sep) (cm/s) | 7±3 | 7±3 | 0.79 |
| E/e’ (sep) | 16.1±7.2 | 16.3±7.1 | 0.80 |
| Deceleration time (ms) | 184±52 | 183±54 | 0.94 |
| Severe AS, n (%) | 22 (4) | 23 (10) | <0.01 |
| Severe AR, n (%) | 6 (1) | 4 (2) | 0.49 |
| Severe TR, n (%) | 84 (15) | 44 (19) | 0.17 |
| MR grades, n (%) |  |  | 0.03 |
| Moderate | 414 (73) | 149 (64) |  |
| Moderate to severe | 80 (14) | 45 (19) |  |
| Severe | 74 (13) | 40 (17) |  |
| ***Treatment for AFMR*** |  |  |  |
| Mitral valve surgery, n (%) | 45 (8) | 50 (21) | <0.01 |
| Mitra Clip, n (%) | 6 (1) | 21 (9) | <0.01 |

Data are expressed as a mean ± SD, median (interquartile range), or number (%). Normally distributed continuous variables were compared using Student’s t-test. Otherwise, continuous variables were compared using a Mann-Whitney U test. Fisher’s exact test was used to analyze categorical variables. Abbreviations: ACE, angiotensin-converting enzyme; ADL, activity of daily living; AF, atrial fibrillation; AFMR, atrial functional mitral regurgitation; AR, aortic regurgitation; ARB, angiotensin II receptor blocker; AS, aortic stenosis; BNP, brain natriuretic peptide; CONUT, Controlling Nutritional Status; COPD, chronic obstructive pulmonary disease; E/e’, early mitral inflow velocity divided by mitral annular early diastolic velocity; ERO, effective regurgitant orifice; GNRI, Geriatric Nutritional Risk Index; IVC, inferior vena cava; IVSd, intraventricular septal thickness at diastole; LA, left atrium; LAVI, left atrial volume index; LV, left ventricle; LVDd, left ventricular end-diastolic diameter; LVDs, left ventricular end-systolic diameter; LVEDV, left ventricular end-diastolic volume; LVEF, left ventricular ejection fraction; LVESV, left ventricular end-systolic volume; MR, mitral regurgitation; MRA, mineralocorticoid receptor antagonist; NYHA, New York Heart Association; PASP, pulmonary artery systolic pressure; PNI, Prognostic nutritional index; RVol, regurgitant volume; TAPSE, tricuspid annular plane systolic excursion; TR, tricuspid regurgitation.

**Supplemental Figures:**


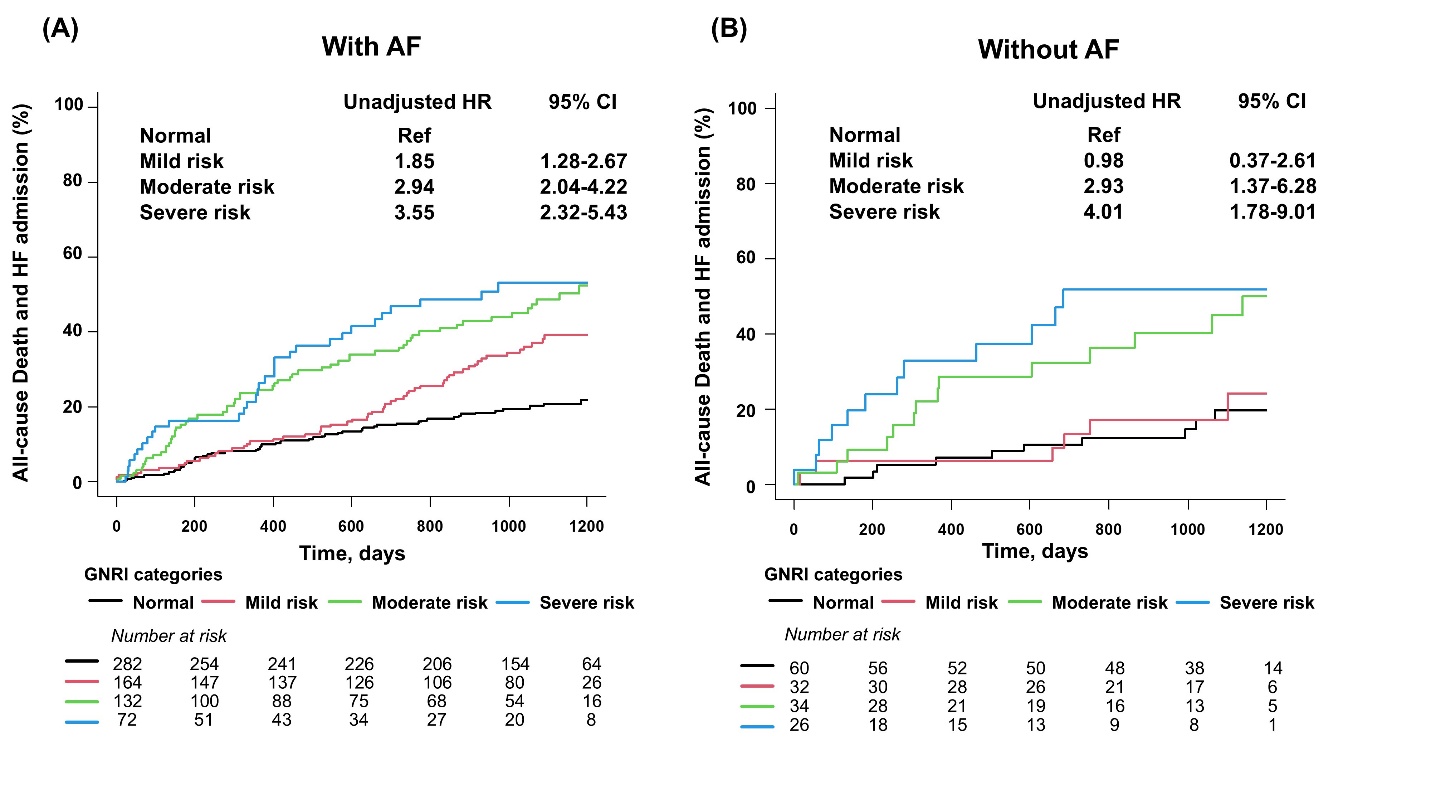


**Supplemental Figure S1.** **Kaplan-Meier curve analysis by the GNRI risk categories in patients with and without AF**

Compared to a normal category, mild, moderate, and severe risk groups assessed by the GNRI were associated with the primary composite outcome of all-cause death and heart failure admission in patients with AF **(A)**. Moderate and severe risk categories were associated with higher rates of the primary outcome compare to the normal category in those with AF **(B)**. AF, atrial fibrillation; CI, confidence interval; GNRI, Geriatric Nutritional Risk Index; HF, heart failure; and HR, hazard ratio.


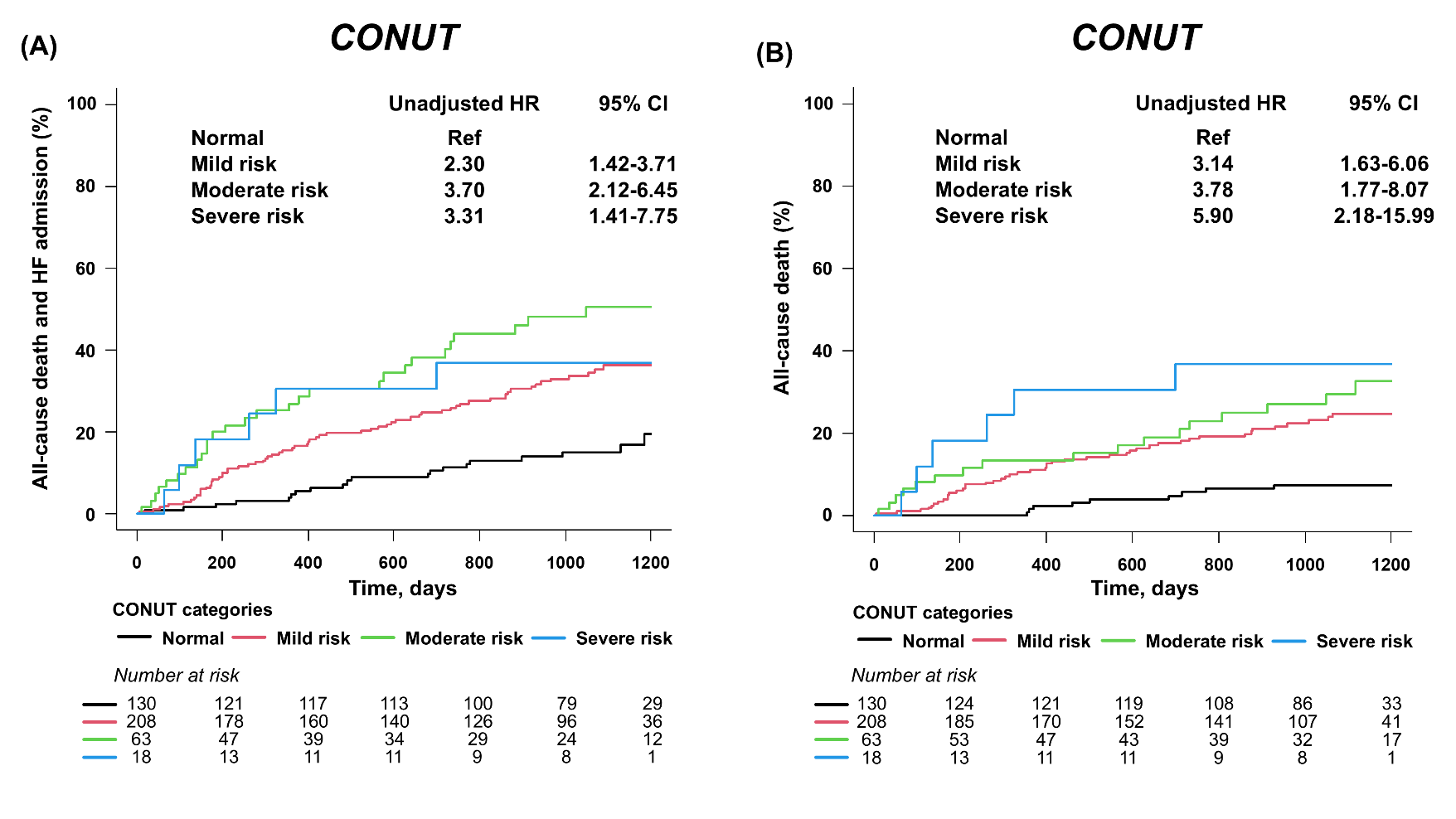


**Supplemental Figure S2. Kaplan-Meier curve analysis by CONUT score**

Compared to a normal category, mild, moderate, and severe risk groups assessed by the CONUT were associated with **(A)** the primary composite outcome of all-cause death and heart failure admission and **(B)** all-cause death. CI, confidence interval; CONUT, Controlling Nutritional Status; HF, heart failure; and other abbreviations as in Supplementary Figure 1.


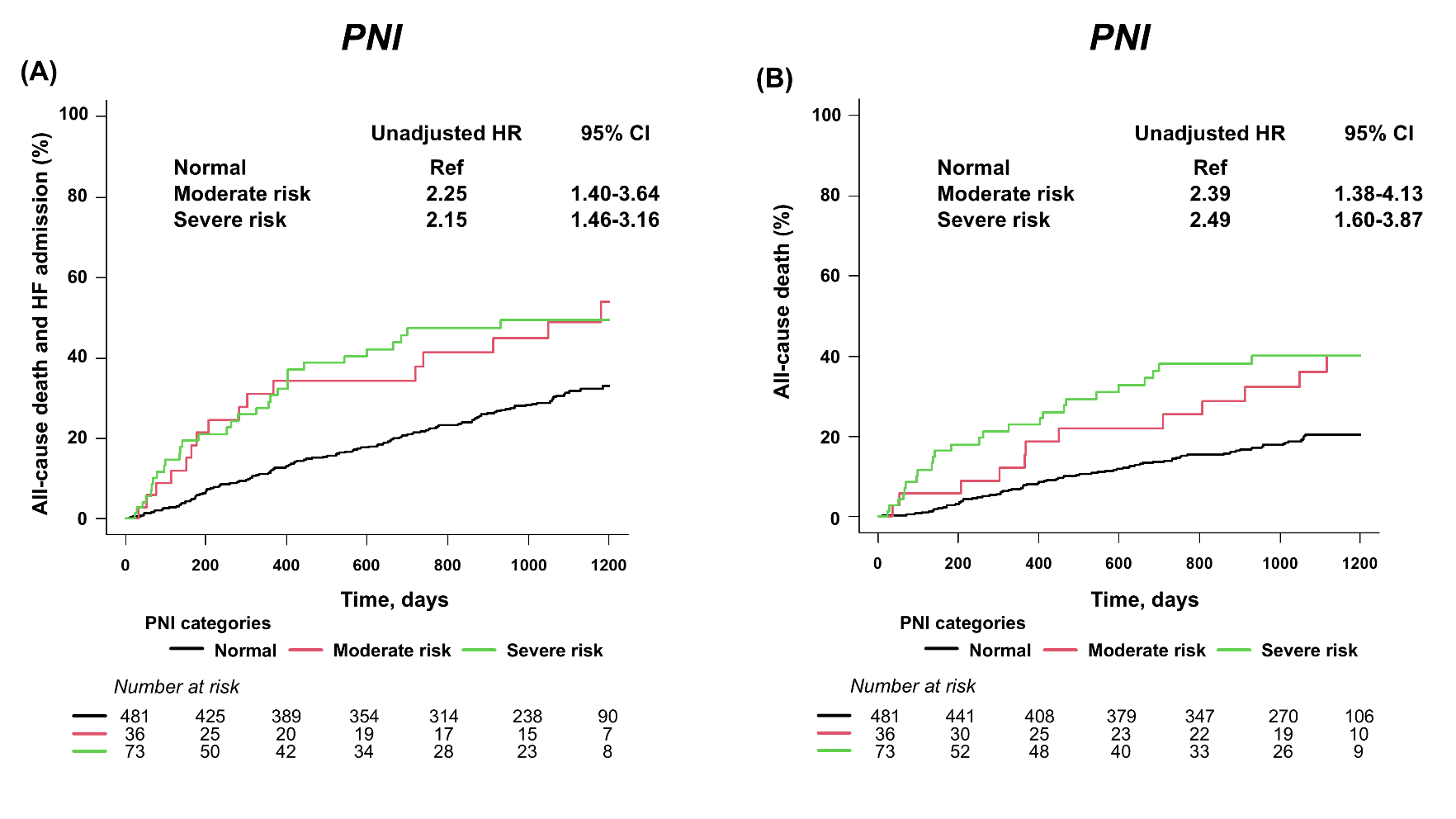


**Supplemental Figure S3. Kaplan-Meier curve analysis by PNI**

Similarly, increasing severity of malnutrition risk assessed by the PNI was associated with **(A)** the primary composite outcome of all-cause death and heart failure admission and **(B)** all-cause death. PNI, Prognostic nutritional index; and other abbreviations as in Supplementary Figure 1.
